# Aminosilane-Functionalized Zeolite Y in Pebax Mixed Matrix Hollow Fiber Membranes for CO_2_/CH_4_ Separation

**DOI:** 10.3390/polym15010102

**Published:** 2022-12-26

**Authors:** Soon-Chien Lu, Thakorn Wichidit, Thanitporn Narkkun, Kuo-Lun Tung, Kajornsak Faungnawakij, Chalida Klaysom

**Affiliations:** 1 Department of Chemical Engineering, Faculty of Engineering, Chulalongkorn University, Bangkok 10330, Thailand; 2 National Nanotechnology Center (NANOTEC), National Science and Technology Development Agency (NSTDA), Pathum Thani 12120, Thailand; 3 Department of Chemical Engineering, National Taiwan University, Taipei 106, Taiwan

**Keywords:** Pebax, zeolite Y, aminosilane treatment, CO_2_/CH_4_ separation, mixed matrix composite, hollow fiber membrane

## Abstract

Due to their interfacial defects between inorganic fillers and polymer matrices, research into mixed matrix membranes (MMMs) is challenging. In the application of CO_2_ separation, these defects can potentially jeopardize the performance of membranes. In this study, aminosilane functionalization is employed to improve the nano-sized zeolite Y (ZeY) particle dispersion and adhesion in polyether block amide (Pebax). The performance of CO_2_/CH_4_ separation of Pebax mixed matrix composite hollow fiber membranes, incorporated with ZeY and aminosilane-modified zeolite Y (Mo-ZeY), is investigated. The addition of the zeolite filler at a small loading at 5 wt.% has a positive impact on both gas permeability and separation factor. Due to the CO_2_-facilitated transport effect, the performance of MMMs is further improved by the amino-functional groups modified on the ZeY. When 5 wt.% of Mo-ZeY is incorporated, the gas permeability and CO_2_/CH_4_ separation factor of the Pebax membrane are enhanced by over 100% and 35%, respectively.

## 1. Introduction

For the production of electricity or the transport of vehicle engines, biogas is considered a viable alternative fuel. The generation of biogas is environmentally friendly; biogas can also be produced by the decomposition of organic compounds through the process of anaerobic digestion, thus reducing organic waste from agriculture, landfills, and industries [1]. Regular biogas consists of 55–70% methane (CH_4_), 30–45% carbon dioxide (CO_2_), and a small amount of water and hydrogen sulfide (H_2_S) [2]. Hence, the removal of excess CO_2_ and corrosive components such as CO_2_ and H_2_S is necessary to improve its heating and market value.

Several conventional technologies, including chemical absorption, pressure swing adsorption, and cryogenic distillation, have been applied for the separation of CO_2_ from biogas. However, due to various drawbacks, e.g., equipment corrosion, chemical contamination, and high energy consumption of such processes, an alternative technique is required. The membrane-based CO_2_ gas separation technique has attracted much attention owing to its low energy consumption, low operating costs, small installation area, and the possibility for industrial scale-up [3]. Separation efficiency and feasibility of the membrane process are highly dependent on the membrane materials.

Because of their simplicity in the process of fabrication, as compared to that of inorganic membranes, polymeric membranes are mostly applied in biogas upgrading [4]. Polyether block amide (Pebax) is a copolymer that consists of polyamide (PA) and polyethylene oxide (PEO). On account of its rigid chemical structure from PA segments, Pebax has good mechanical stability [5]. Low-cost Pebax can also provide outstanding CO_2_ permeability due to the dipole–quadrupole interaction between the CO_2_ and PEO segments in the polymer structure [6]. Pebax has thus gained increasing attention as one of the promising materials for the application of CO_2_ gas separation [7]. Several attempts to improve the CO_2_/CH_4_ selectivity of Pebax so that it can be implemented in CO_2_/CH_4_ separation, especially in biogas upgrading, have currently been reported [7,8]. The performance of the polymeric membranes can be improved by the addition of the following inorganic phases viz. zeolite, carbon molecular sieve (CMS), silica particle, metal oxide, carbon nanotube (CNT), metal–organic framework (MOF), and graphene into the polymer matrix to form mixed matrix membranes (MMMs) [8]. 

Zeolites are aluminosilicate inorganic materials whose framework contains AlO_4_ and SiO_4_ tetrahedral linked to each other by the sharing of oxygen atoms in each corner [9]. As such, zeolites have well-defined porous structures at the angstrom level and can be utilized as a kind of molecular sieve to differentiate between molecules of different sizes and shapes for the purpose of separation, based on the size of their specific pore [10]. There are different types of zeolites with distinctive internal pore structures such as ZSM-5, zeolite beta, zeolite A, and zeolite Y. Of the several types of zeolite, zeolite Y, a faujasite (FAU) framework type, has been demonstrated to be a good candidate as a filler for MMMs in CO_2_/CH_4_ separation. Zeolite Y offers a large window size without steric hindrance for gas transport and provides good electrostatic quadrupole interaction with CO_2_. Zeolite Y has been incorporated with glassy polymers such as polyimide and was found to significantly improve CO_2_/CH_4_ selectivity (>55%) without diminishing the gas permeance [11,12]. However, the addition of inorganic fillers into a polymer matrix often typically causes a kind of defective “interfacial void” as a result of poor material compatibility. This defectiveness decreases the gas selectivity of the membranes and often results in degradation of the overall performance of membrane gas separation [13]. To further enhance the adsorption capability and selectivity of ZeY, it may be necessary to integrate an organic functional group onto its surface through modification with aminosilane or ethylenediamine [14]. Studies have shown that aminosilane functionalization of inorganic fillers can lead to better interaction with the polymer matrix [15]. Moreover, the amino group in the silane coupling agent possesses high affinity with CO_2_, thus further improving the gas selectivity of resultant membranes [16,17].

As yet, incorporating ZeY with Pebax to form MMMs, for the application of CO_2_/CH_4_ separation, has not been widely explored. Previous works regarding MMMs focused solely on flat-sheet membranes in single-dense film configuration owing to its less complicated fabrication compared to hollow fiber or composite configurations. In this work, MMMs from Pebax and nano-sized ZeY filler have been fabricated in hollow fiber form; a selective layer of MMMs having a thickness less than 6 µm was coated on polysulfone (PSF) hollow fiber support. In addition, N-[3-(trimethoxysilyl)propyl]ethylenediamine (AEAPTMS) was used to modify the external surface of ZeY in order to improve the interaction between the filler and the polymer as well as the dispersion of the filler in the polymer matrix. The effect of the modification of the ZeY surface on the performance of membrane gas separation was also investigated. 

## 2. Materials and Methods

### 2.1. Materials

**Figure 1:**
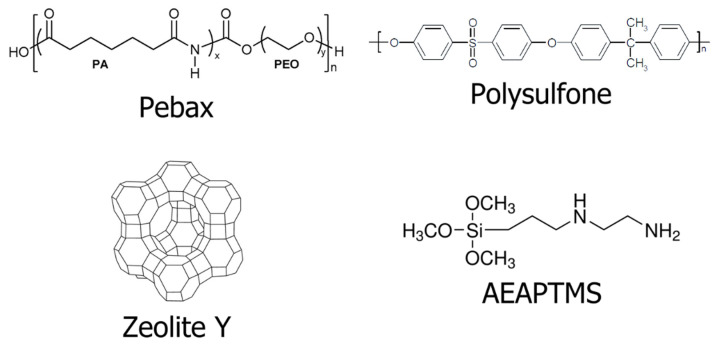
Schema of the chemical structure of Pebax, polysulfone, zeolite Y, and N-[3-(trimethoxysilyl)propyl]ethylenediamine (AEAPTMS) used in this study.

Polysulfone (PSF, with a molecular weight of ~22,000 g/mol) and N-[3-(trimethoxysilyl)propyl]ethylenediamine (AEAPTMS, with a molecular weight of ~222.36 g/mol, 97%) were purchased from Sigma-Aldrich (St. Louis, MO, USA). Polyether block amide (Pebax 1657) was bought from Arkema Inc. (Colombes, France). *N*-methyl-2-pyrrolidone (NMP, 99%) was acquired from Acros Organics (Waltham, MA, USA). Ethanol (EtOH) was procured from Carlo Erba (Cornaredo, Italy). Zeolite Y-type powder (ZeY, CBV720) with 250 nm particle size and 15 silicon:aluminium mole ratio (Si/Al) was provided by Zeolyst International (Conshohocken, PA, USA). In Figure 1, the chemical structures of Pebax, polysulfone, ZeY, and AEAPTMS are shown.

### 2.2. Zeolite Y Surface Modification

The ZeY powder was heated in a vacuum oven at 100 °C overnight to remove moisture before the process of grafting. Then, 2 g of dried ZeY powder and 100 mL of EtOH were added into a round bottom flask; the mixture was sonicated for 30 min. Next, the slurry was heated by stirring at 85 °C for 1 h. AEAPTMS (8 mL) was then poured into the pre-heated mixture for amino grafting under reflux of nitrogen gas for 24 h. The surface-modified zeolite Y (Mo-ZeY) was cooled down to ambient temperature, filtered, and washed by a polar medium several times. Finally, the resultant Mo-ZeY was heated in a vacuum oven at 100 °C overnight to remove the excess solvent.

### 2.3. Fabrication of the Hollow Fiber Support

To prepare the homogeneous dope solution with 20 wt.% of polymer, both PSF and NMP were mixed and stirred at 65 °C. Before extrusion, the prepared dope solution was degassed via sonication for 30 min. Distilled water was used as a bore fluid and was co-extruded with the PSF solution through a spinneret by a dry-jet/wet quench spinning process. The dope and the bore flow rate were controlled at 1.1 g/min and 0.28 g/min, respectively. The distance of the air gap between the spinneret head and the coagulation bath was fixed at 10 cm. The obtained hollow fiber support was soaked in a water bath at room temperature for 48 h and then dried overnight in a vacuum oven at 50 °C to remove the excess solvent.

### 2.4. Fabrication of the Selective Layer

To fabricate the selective layer on top of the PSF hollow fiber support prepared in the previous sub-section, a dip coating method was applied. Both Pebax pellets and solvent (EtOH to distilled water with 70:30 weight ratio) were added into a beaker, stirred, and heated at 65 °C to prepare a 5 wt.% membrane solution; a homogenous solution was obtained. Accordingly, a desired amount of the filler was added to the solution and stirred to form a slurry. PSF hollow fiber supports were then dipped into the slurry for 15 min. Finally, the coated composite hollow fiber membranes were dried in a vacuum oven at 50 °C overnight.

### 2.5. Characterization of Filler and Membranes

The specific surface area and porosity of ZeY and Mo-ZeY were characterized using physisorption analyzer (BELSORP-max, MicrotracBEL, Osaka, Japan) via N_2_ adsorption–desorption isotherms under liquid nitrogen at 76 K.

X-ray diffraction (ZXRD, Bruker D8 Advance, Billerica, MA, USA) analysis was employed to characterize the crystal structures of the prepared fillers and membranes, with copper (Cu) emitting λ-rays radiation at wavelength 1.54 Å under 40 kV of accelerating voltage and 40 mA of electric current. A scan angle (2θ) was recorded from 5° to 40° with an increment of 0.02 ° s^−1^.

Attenuated total reflectance-Fourier transform infrared spectroscopy (ATR-FTIR, Nicolet 6700, Thermo Scientific, Waltham, MA, USA) was chosen to measure the chemical functional group given that most of the samples in this study were coated hollow fiber membranes. For each specimen, 32 scans were collected having a wavenumber resolution of 4 cm^−1^.

Morphological and structural studies of the fillers and prepared membranes were achieved by scanning electron microscopy (SEM, Hitachi S-3400N, Tokyo, Japan). Liquid nitrogen was used to fracture the membrane samples, which were then coated with gold (Au) by an ion sputter coater (Hitachi E-1010, Tokyo, Japan) before subjecting to SEM analysis.

A glass transition temperature (Tg) of membrane samples was also determined by differential scanning calorimetry (DSC, Shimadzu DSC 60A Plus, Kyoto, Japan) from −100 to 250 °C with 10 °C/min heating rate in N_2_ ambient.

### 2.6. Membrane Performance Test

Hollow fiber membranes were packed in a stainless-steel module having a 40 mm inside diameter using epoxy glue. The membrane module was then attached to a custom-built gas permeation apparatus. Next, a 50:50 mole ratio of CO_2_/CH_4_ gas mixture was fed from the shell side of the module having a flow rate of 20 cm^3^/min. In contrast, helium was fed from the tube side to act as a carrier gas. The whole setup was connected to gas chromatography (GC, Shimadzu GC-14B, Kyoto, Japan) and a film flow meter (Horiba Stec VP-2, Kyoto, Japan). All prepared nanocomposite membranes were tested at steady state and isothermal conditions. The range of temperature and transmembrane pressure was set to 30–70 °C and 1-5 bar, respectively.

The permeability of CO_2_ and CH_4_ was determined by the following equation:

P𝒾 = J𝒾 l/(p_𝒾,0_ − p_𝒾,x_) (1)

where P𝒾 is component 𝒾 gas permeability, J𝒾 is component 𝒾 gas flux, p_𝒾,0_ − p_𝒾,x_ is transmembrane pressure of component 𝒾, and l is the membrane thickness.

Gas permeability was calculated in Barrer unit, where 1 Barrer = 10^−10^ cm^3^(STP).cm/cm^2^.s.cmHg. The gas separation factor (α_𝒾j_) of the membrane is defined as:

α_𝒾j_ = (y_𝒾_/y_j_)/(x_𝒾_/x_j_) (2)

where y and x are the mole fraction in permeate and retentate sides, respectively. The subscripts 𝒾 and j represent the comparative components: CO_2_ and CH_4_ in this work.

## 3. Results

### 3.1. Characterization of Fillers

**Figure 2:**
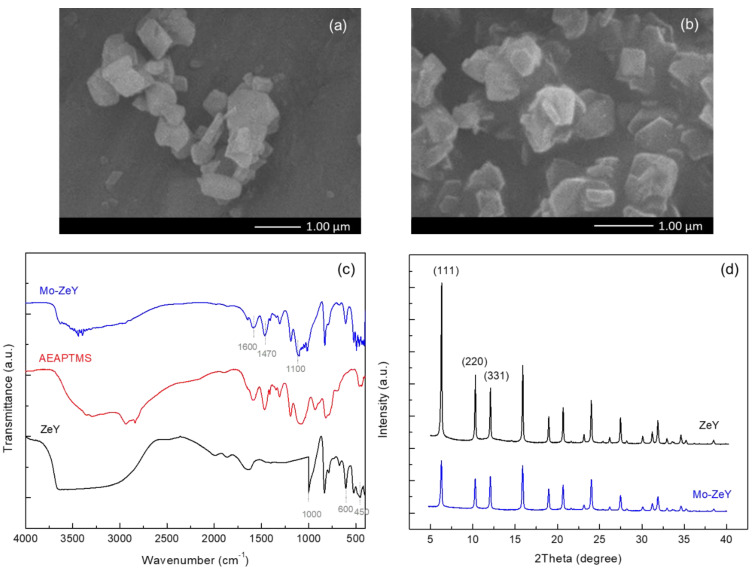
SEM images of (**a**) Pristine zeolite Y (ZeY) and (**b**) Modified zeolite Y (Mo-ZeY) with aminosilane and their corresponding (**c**) ATR-FTIR spectra and (**d**) XRD patterns.

In Figure 2a,b, the morphology of ZeY and Mo-ZeY was investigated. The average particle size of the zeolite with and without surface modification was about 506 nm and 509 nm, respectively, a considerably insignificant change in particle size from the pristine zeolite. In Figure 2a, the morphology of ZeY particles is clearly defined. In Figure 2b, the Mo-ZeY particles are seen to be stacked on top of each other, which may be the result of introducing the aminosilane coupling agent onto the zeolite, thus enhancing the interfacial adhesion between the particles [18].

Attenuated total reflectance-Fourier transform infrared spectroscopy (ATR-FTIR) was used to test the above postulation that aminosilane was successfully introduced into the system. N-[3-(trimethoxysilyl)propyl]ethylenediamine (AEAPTMS) was selected as a grafting agent in this study. In Figure 2c, the FTIR spectra of ZeY and AEAPTMS are displayed. A peak was observed at 450 cm^−1^ that indicated Si-O-Al stretching of ZeY. The two peaks at 600 and 1000 cm^−1^ were contributed by the internal and external tetrahedral symmetrical stretching of Si-O. The broad peak at around 3000–3600 cm^−1^ is assigned to the hydroxyl group (O-H) in the sodalite cage in the ZeY structure [19].

In Figure 2c, the FTIR spectrum of Mo-ZeY is shown. The two peaks at 1470 and 1600 cm^−1^ were assigned to N-H bending and -NH_2_ scissoring from AEAPTMS, respectively. In this reaction, the silanol group (Si-O-H) of aminosilane underwent self-condensation and reacted with the hydroxyl groups of the zeolite surface, forming a siloxane bond (Si-O-Si) on the zeolite surface [20]. Thus, the bands at about 1030, 1055, 1100, and 1150 cm^−1^ were referred to as siloxane bonds and siloxane linkage due to the grafting reaction [21]. The two peaks at 3350 and 3410 cm^−1^ were caused by the primary amine (-NH_2_) from AEAPTMS. On the other hand, the broad peak from the vibration of the hydroxyl group was reduced due to stretching of the primary amine absorption at the same frequency (at around 3300–3500 cm^−1^) [22,23]. This outcome suggests that AEAPTMS was successfully grafted on the zeolite surface.

In Figure 2d, the XRD patterns of zeolite Y with and without surface modification are shown. The characteristic peaks at 2θ = 6.3°, 10.3°, 12°, 15.9°, 19°, 20.7°, 24°, 27.5°, and 32.1° were identified for both zeolite samples [22]. This observation indicates no considerable change in the structural property of the zeolites after silylation. Nevertheless, the intensity of the three peaks in Mo-ZeY at 2θ = 6.3, 10.3, and 15.9°, corresponding to (111), (220), and (331) lattice planes of the faujasite (FAU) framework, was significantly reduced [24]. Such an outcome may be due to new bonds formed between Si from the grafting agent and O from the surface and/or channels of the ZeY [9]. The schematic diagram showing the silylation reaction on the surface of zeolite is provided in Figure S1. These findings also indicate the completion of aminosilane grafting onto the ZeY surface.

In Table 1, the porosity properties of ZeY before and after grafting are summarized. As expected, other than the particle size that increased very slightly after aminosilane treatment, the BET surface area, pore volume, and pore diameter of ZeY were all reduced after surface modification. This outcome was caused by AEAPTES grafted on the pore-wall of ZeY. As such, the porosity properties of ZeY were consistent with the XRD characteristics mentioned previously, i.e., modification of the ZeY surface resulted in a narrowing of its particle pores. A similar observation was reported by Sanaeepur et al. [9]. 

### 3.2. Membrane Characterization 

**Figure 3:**
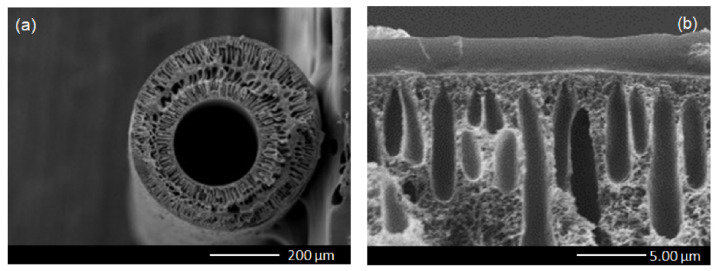
Cross-sectional view of (**a**) PSF hollow fiber support, and (**b**) PSF hollow fiber support coated with Pebax.

In Figure 3, the cross-sectional morphology of the PSF hollow fiber support with and without Pebax coating was examined. The PSF hollow fiber support was presented with a finger-like porous structure at the bottom and a dense layer at the outer surface. Both the outside diameter and thickness of the PSF hollow fiber support were about 650 μm and 140 μm, respectively. Pebax was coated on top of the hollow fiber support and formed a selective layer having a thickness of 2.62 μm.

**Figure 4:**
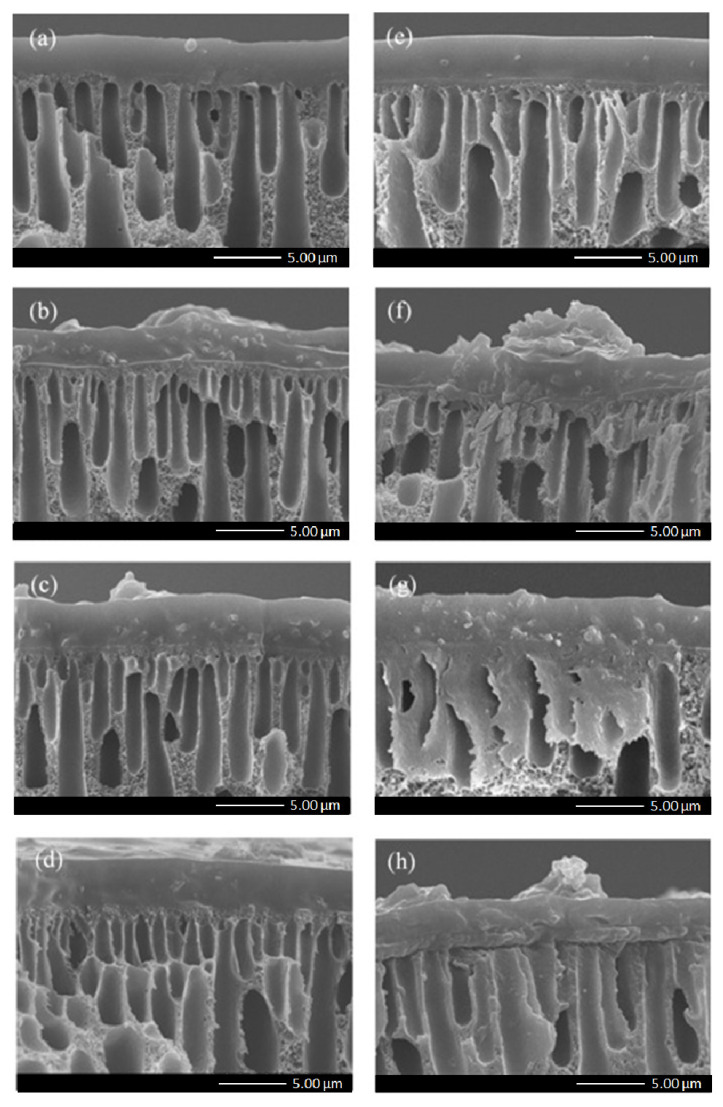
Cross-sectional view of MMMs incorporated with (**a**) 5, (**b**) 10, (**c**) 15, and (**d**) 20 wt.% of ZeY, respectively, and with (**e**) 5, (**f**) 10, (**g**) 15, and (**h**) 20 wt.% of Mo-ZeY, respectively.

**Figure 5:**
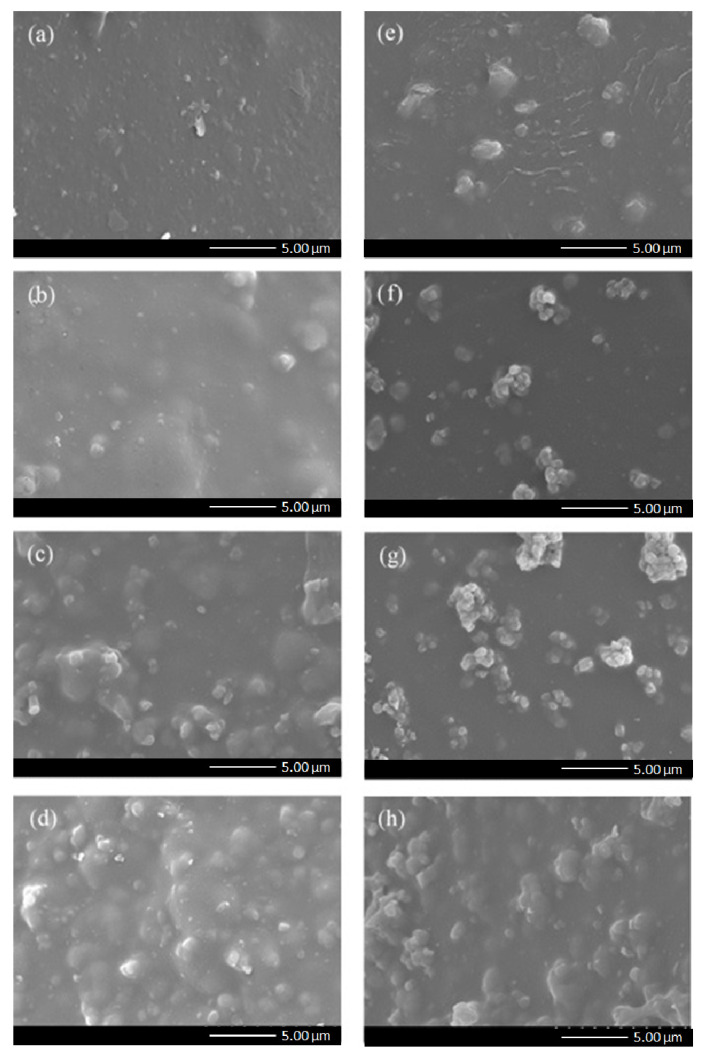
Top view of MMMs incorporated with (**a**) 5, (**b**) 10, (**c**) 15, and (**d**) 20 wt.% of ZeY, respectively, and with (**e**) 5, (**f**) 10, (**g**) 15, and (**h**) 20 wt.% of Mo-ZeY, respectively.

ZeY or Mo-ZeY was then added into the Pebax matrix of the selective layer to form MMMs and improve the performance of gas separation. The effect of fillers on the membrane morphology and properties was also inspected via SEM. MMMs have a Pebax continuous phase and a dispersal phase of inorganic filler. In Figure 4 and Figure 5, the cross-sectional view and top surface morphology of all MMMs prepared in this study are shown. The top layer of all samples is dense and integrally coated onto the PSF hollow fiber support without any observable interfacial void between the filler and polymer. The average thickness and cross-sectional surface area of the selective layer slowly increased with increasing filler loading (please refer to Table S1 in the supplementary material). It is noted that when more fillers were added to the Pebax matrix, it was more difficult to achieve a uniform coating on the surface.

In Figure 5, when the particles were incorporated into the Pebax phase, both ZeY and Mo-ZeY showed good dispersion. However, at 15 wt.% loading, ZeY started to agglomerate, while for its counterpart Mo-ZeY, agglomeration occurred at 10 wt.% loading. Such an outcome may be caused by the higher interparticle affinity possessed by Mo-ZeY after aminosilane grafting, making the particles more susceptible to agglomeration at lower loadings, as compared to pristine ZeY [25]. Nonetheless, no interfacial gap and void between the dispersed and continuous phases of MMMs were observed in all samples.

**Figure 6:**
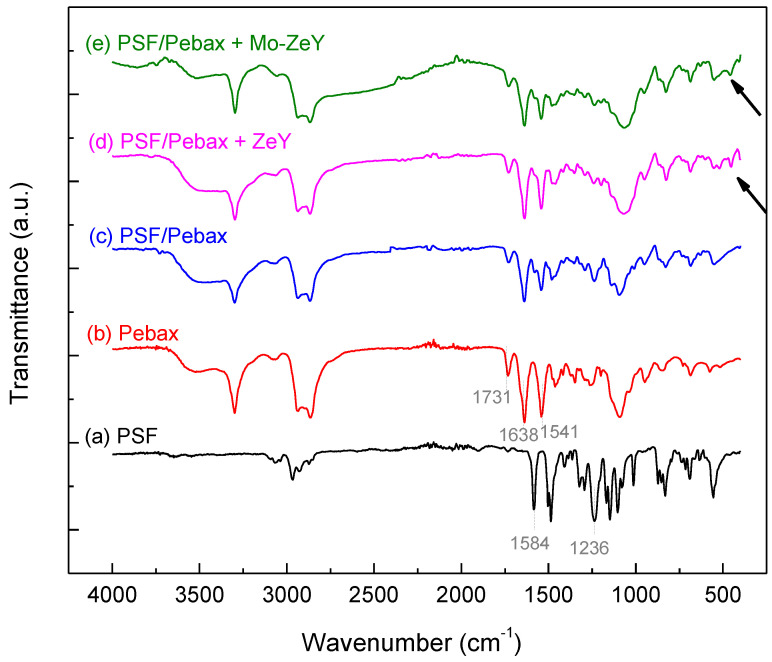
ATR-FTIR spectra of (**a**) PSF support, (**b**) Pristine Pebax, (**c**) PSF/Pebax composite membrane, (**d**) PSF/Pebax + 20 wt.% ZeY MMM, and (**e**) PSF/Pebax + 20 wt.% Mo-ZeY MMM. The two arrows indicate where new peaks are formed when ZeY or Mo-ZeY is incorporated with Pebax.

In Figure 6, the ATR-FTIR spectra of the PSF hollow fiber support, pristine Pebax, PSF/Pebax composite membrane, and PSF/Pebax with 20 wt.% ZeY/Mo-ZeY MMMs are shown. This is to verify several functional groups of the prepared membranes and their components. Three prominent peaks of PSF were observed at 1584, 1236, and 1147 cm^−1^ corresponding to C=C conjugation in the aromatic ring, C-O between aromatic rings, and S=O symmetric in a sulfonate group, respectively [26]. Further, several major stretching vibrations in the Pebax membrane were observed at 3300–3400, 1638, 1541, and 1731 cm^−1^ indicating secondary amine (=N-H) and N-H region, amide bonding (-CONH), carbonyl group (C=O) in polyamide (PA) section, and carbonyl group (C-O) in polyethylene oxide (PEO) section, respectively [27].

In Figure 6, the FTIR spectra of Figure 6c–e reveal the same characteristic peaks as those of the pristine Pebax in Figure 6b. Thus, it is seen that Pebax was successfully coated on the PSF hollow fiber support. Moreover, a small peak was observed at 450 cm^−1^ in MMMs with both ZeY and Mo-ZeY corresponding to the new bonding with ZeY, as indicated by the two arrows in Figure 6d,e.

To trace the change in the crystalline property of the polymer matrix after incorporating the fillers, XRD analysis was carried out. Moreover, a selective layer was coated onto the glass slide and subjected to XRD. Subsequently, the selective layer was scraped off from the glass slide and sent for DSC analysis to determine the glass transition temperature (Tg). The percentage of the crystalline degree of each membrane specimen can be expressed as follows [28]:

Crystalline degree = [X_C_/(X_C_ + X_A_)] × 100 (3)

where X_C_ and X_A_ are the area under the crystalline and the amorphous region in the XRD pattern, respectively.

**Figure 7:**
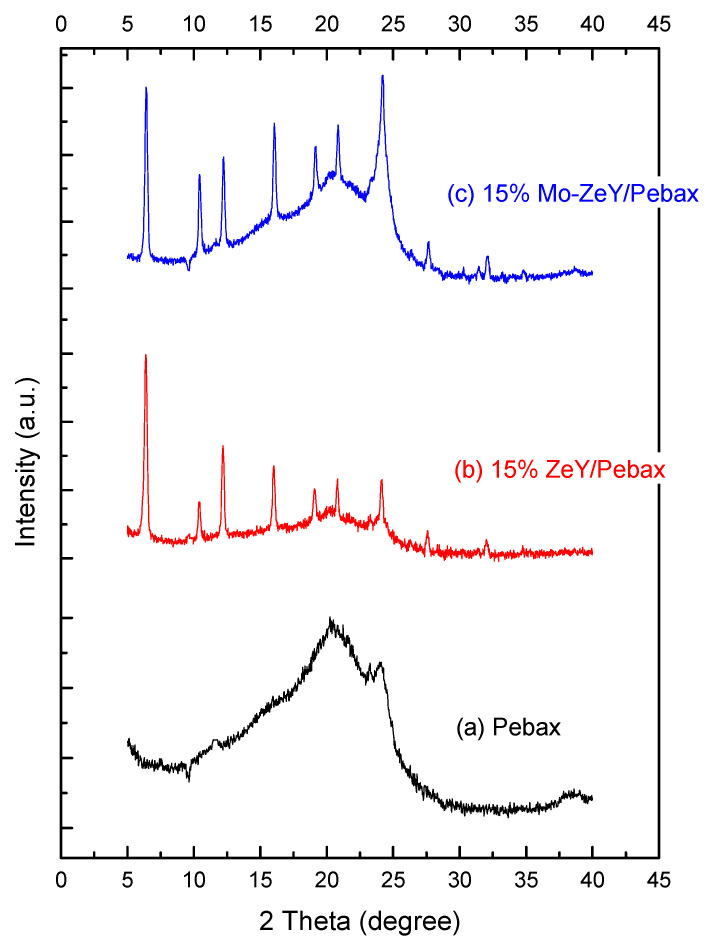
XRD patterns of (**a**) Pebax, (**b**) Pebax + 15 wt.% ZeY, and (**c**) Pebax + 15 wt.% Mo-ZeY membranes.

In Figure 7, XRD patterns of Pebax in comparison to Pebax with ZeY and Mo-ZeY at 15 wt.% are depicted. Pebax has a semi-crystalline structure, comprising a mixture of crystalline and amorphous regions from PA and PEO, respectively. The sharp peak at 2θ = 24.0° refers to the crystalline structure; the broad peaks from 13.7° to 21.0° refer to the amorphous section of Pebax [29].

In Table 2, both glass transition temperature (Tg) and degree of crystallinity of the membranes are enhanced owing to the increasing amount of filler loading. The increased Tg and crystallinity may have arisen due to the formation of H-bonds between the filler surface and polymer chain, consequently restricting the mobility and flexibility of the Pebax chain [30]. Furthermore, the addition of fillers affected the packing of the surrounding polymers by forming a more ordered arrangement, resulting in an increase in crystallinity [31,32]. 

### 3.3. Gas Separation Performance of Membranes

Gas separation properties in a polymeric material are influenced by free volume in membrane structures as well as the interaction between penetrant gases and polymers [33]. As mentioned previously, Pebax used in this study is a copolymer of PA and PEO blocks. The crystalline PA segments provide mechanical support to membranes while its amorphous counterpart from PEO segments offers flexible transport pathways to target gas molecules. The presence of the carbonyl group in PEO and the amine group in PA also helps to promote the permeation of CO_2_ via dipole-induced dipole force [34]. The gas transport in the membranes can be enhanced by the presence of a zeolite framework [9,10].

**Figure 8:**
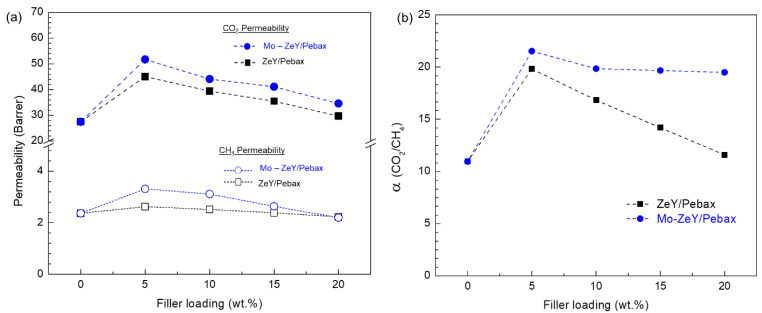
(**a**) Gas permeability and (**b**) CO_2_/CH_4_ separation factor of MMMs with ZeY or Mo-ZeY at various filler loadings. The membranes were tested at 1 bar of transmembrane pressure and 30 °C.

In Figure 8, CO_2_ and CH_4_ permeability and CO_2_/CH_4_ separation factor of the prepared membranes with different filler loadings from ZeY or Mo-ZeY are presented. The membranes were tested with a gas mixture of CO_2_ and CH_4_ at 50/50 molar ratio at 1 bar of transmembrane pressure and 30 °C. Applying the same filler loading, MMMs with Mo-ZeY showed a better separation performance compared to ZeY/Pebax membranes, since the amino group grafted onto the surface of zeolite Y particles enhanced their interaction with CO_2_ molecules. Moreover, RNH_2_ (primary amine) on the Mo-ZeY surface is seen to react with CO_2_ to form RNHCO_2_^−^ (carbamate ions) that facilitated the transport of CO_2_ molecules by solution and diffusion through the matrix of Pebax [22].

The gas permeability and separation factor of MMMs from both ZeY and Mo-ZeY tended to significantly increase at a small filler loading (5 wt.%) and dropped step-by-step at higher loadings. Such a result may be because the fillers started to agglomerate and form a non-selective path via intrapore among the aggregated particles at higher loadings. In addition, the excess filler addition in the Pebax matrix can disturb the polymer chain mobility and reduce flexibility in the backbone [35]. A decrease in gas permeability may also be related to the rigidification of the polymer matrix due to the consistent increase in crystallinity at higher filler loadings. The increase in Tg values in the previous section also verifies the signs of rigidification in the Pebax polymer matrix [36]. In this study, the experimental results suggest that 5 wt.% of filler is the best loading option, exhibiting good compatibility between Pebax and fillers and providing the highest membrane performance with effective particle dispersion. The addition of a suitable filler into the polymer matrix can enhance its membrane performance, but only to a certain extent. To uncover the appropriate filler loading for an MMM, specifically at lower filler loadings, a more detailed study should be carried out.

**Figure 9:**
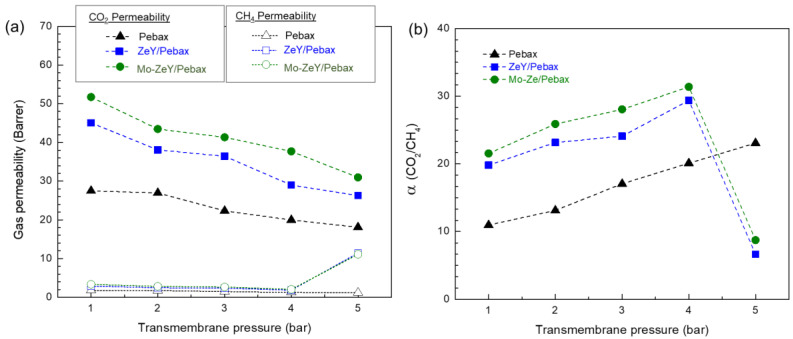
(**a**) Gas permeability and (**b**) CO_2_/CH_4_ separation factor of neat Pebax, MMMs with 5 wt.% of ZeY and Mo-ZeY tested at 30 °C, and 1 to 5 bar of transmembrane pressure.

In Figure 9, the gas permeability and CO_2_/CH_4_ separation factor of the neat Pebax and MMMs with 5 wt.% of ZeY and Mo-ZeY, tested at 30 °C, and 1 to 5 bar of transmembrane pressure, are displayed. When transmembrane pressure increased from 1 to 4 bar, the permeability of CO_2_ and CH_4_ for all three samples reduced, but the CO_2_/CH_4_ separation factor improved. Zhang et al. reported similar observations when an aminosilane-functionalized graphene oxide was incorporated into the Pebax membrane [37]. In fact, the observed trend of decreasing gas permeability along with increasing upstream pressure is a common behavior in a glassy polymer at relatively low pressure below 30 bar [38,39]. Such behavior is well explained by a dual-mode model whereby the sorption sites or “microvoids” between polymer chains become saturated at higher penetrant concentration (or higher pressure in our case), resulting in a decrease in gas permeability [39]. Nonetheless, this behavior can affect the transportation of CH_4_ with a larger kinetic diameter (0.38 nm) more than CO_2_ (0.33 nm) [40]. As a result, when the transmembrane pressure is increased, CO_2_/CH_4_ gas separation factor is enhanced.

When transmembrane pressure was further increased to 5 bar, CH_4_ permeability of membranes with ZeY and Mo-ZeY increased dramatically. In contrast, their corresponding CO_2_ permeability continued to decrease. In Figure 9b, a radical fall in CO_2_/CH_4_ gas separation factor is seen. As the neat Pebax membrane sample was unaffected under the same testing conditions, it can be deduced that such a difference was caused by the zeolite filler. Both Khalkhali et al. and Zhang et al. have reported similar observations from their molecular simulation studies; a sudden surge of CH_4_ sorption was observed at around 500 kPa or 5 bar for faujasite-type zeolites, such as zeolite X and zeolite Y [41,42]. At pressure above 5 bar, the adsorption of CH_4_ became more competitive, while CO_2_ sorption almost reached the sorption capacity of the zeolite. Therefore, at this point, the increase in CH_4_ adsorption in the zeolite was far more pronounced; CO_2_/CH_4_ separation factor thus dropped. 

**Figure 10:**
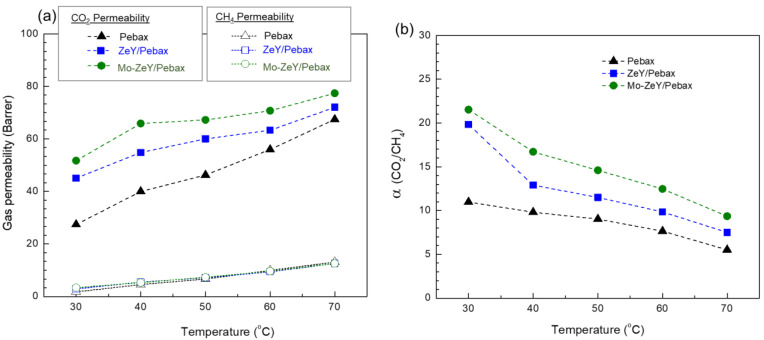
(**a**) Gas permeability and (**b**) CO_2_/CH_4_ separation factor of neat Pebax and MMMs with 5 wt.% of ZeY and Mo-ZeY tested at 1 bar of transmembrane pressure and 30 to 70 °C.

In Figure 10, both gas permeability and CO_2_/CH_4_ separation factor of neat Pebax and MMMs with 5 wt.% of ZeY and Mo-ZeY were tested at different temperatures with 1 bar of transmembrane pressure. When the operating temperature was raised from 30 to 70 °C, CO_2_ and CH_4_ permeability increased, but CO_2_/CH_4_ separation factor decreased for all samples. It is seen that the increased thermal energy caused the polymer chain to become more flexible, thus creating a higher free volume that promoted the transport of CO_2_ and CH_4_ [43,44]. 

In Table 3, performance of the membrane developed in this work was compared with the membrane based on the same polymer matrix having different types of zeolite fillers, as reported in the literature. 

One should keep in mind that the membrane developed in this work was tested with a gas mixture at high CO_2_ content that is well known to obtain a lower separation factor in comparison when tested with single gas. In addition, the membrane from this work was fabricated in a composite hollow fiber configuration having a relatively thinner selective layer (around 3–6 µm) compared to a dense flat-sheet membrane having a thickness of more than 20 µm. The CO_2_ permeability of the dense flat-sheet membrane in Barrer, which is proportional to the membrane thickness, is thus higher than the membrane developed via this work.

## 4. Conclusions

In this study, the addition of zeolite fillers (ZeY and Mo-ZeY) provided a substantial improvement to overall membrane performance when they were incorporated into the Pebax matrix as a selective layer coated on the PSF hollow fiber membranes. Despite the problem of particle agglomeration in high filler loadings, the performance of Mo-ZeY/Pebax MMMs was far superior in comparison to its ZeY counterpart in almost every category of testing performed in this study, including gas permeance and separation factor over a range of filler loadings, temperatures, and transmembrane pressures. It is seen that aminosilane grafted onto the ZeY surface had beneficial effects on CO_2_ gas separation. Operating pressure and temperatures also had a significant impact on the performance of membrane gas separation. For all membrane samples, raising transmembrane pressure resulted in the reduction of gas permeability, but CO_2_/CH_4_ separation factor increased. In contrast, when operating temperature increased from 30 to 70 °C, gas permeability increased, but CO_2_/CH_4_ separation factor decreased in all membranes. 

## Figures and Tables

**Table 1:** The physical properties of ZeY and Mo-ZeY.

Sample	Average Particle Size (nm)	BET Surface Area (m^2^/g)	Total Pore Volume (cm^3^/g)	Average Pore Diameter (nm)
ZeY	506	750	0.40	2.32
Mo-ZeY	509	305	0.20	1.88

**Table 2:** Glass transition temperatures (Tg) and calculated degree of crystallinity of prepared MMMs with different ZeY and Mo-ZeY loadings.

Samples	Filler Loading (wt.%)	Tg (°C)	Crystallinity (%)
Pebax	0	−54.8	10.2
ZeY/Pebax	5	−54.6	10.4
	10	−54.1	14.6
	15	−53.6	15.3
	20	−52.6	18.9
Mo-ZeY/Pebax	5	−54.5	10.9
	10	−54.0	14.9
	15	−53.4	15.5
	20	−51.8	19.0

**Table 3:** Performance comparison of the membrane based on Pebax-1657 with different types of zeolite filler.

Membrane		Testing Conditions			Performance		Ref.
Filler	Membrane Type	Temperature (°C)	Pressure (bar)	Feed Gas Compositions (CO_2_/CH_4_)	Separation Factor	CO_2_ Permeability (Barrer)	
Modified zeolite Y	Composite hollow fiber	30	5	Mixed gas 50/50	31.4	37.7	This work
Si-CHA zeolite	Flat-sheet	Ambient	5	Mixed gas 30/70	20.1	73.3	[45]
ZSM-5 zeolite	Flat-sheet	35	3	Single gas	25.1	251.5	[46]
Modified SAPO-34	Flat-sheet	25	2	Mixed gas 50/50	34.7	137.1	[47]
COOH-functionalized NaX	Composite flat-sheet	25	6	Single gas	57.4	187.76	[48]
Zeolite NaX	Flat-sheet	Ambient	8	Single gas	45	95	[49]

## Data Availability

Not applicable.

## Supplementary Materials

The following supporting information can be downloaded at: https://www.mdpi.com/article/10.3390/polym15010102/s1. Figure S1. Silylation reaction of AEAPTMS on the surface of zeolite Y.; Table S1. Average thickness and surface area estimated from the cross-sectional view of MMMs.
