# Supplementary material for: Aminosilane-Functionalized Zeolite Y in Pebax Mixed Matrix Hollow Fiber Membranes for CO2/CH4 Separation
Source: Polymers (Basel). 2022 Dec 26;15(1):102. doi: 10.3390/polym15010102 (PMC9823953; doi:10.3390/polym15010102)
Supplement: Supplementary file 1 [file polymers-15-00102-s001.zip › polymers-2100288-supplementary.pdf]

Supplementary Material

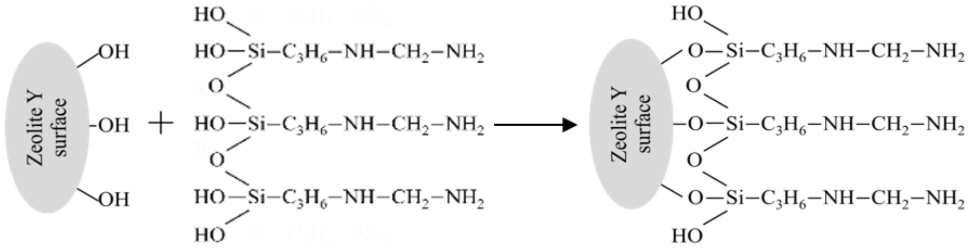

Figure S1. Silylation reaction of AEAPTMS on the surface of zeolite Y.

Table S1. Average thickness and surface area estimated from cross-sectional view of MMMs.

Samples	Thickness (μm)	Surface Area (cm^2)
Pebax + 5 wt.% of ZeY	2.83±0.25	6.179
Pebax + 10 wt.% of ZeY	2.94±0.31	6.182
Pebax +15 wt.% of ZeY	3.05±0.24	6.184
Pebax + 20 wt.% of ZeY	3.09±0.26	6.184
Pebax + 5 wt.% of Mo-ZeY	3.16±0.17	6.186
Pebax + 10 wt.% of Mo-ZeY	3.16±0.86	6.186
Pebax + 15 wt.% of Mo-ZeY	3.30±0.25	6.188
Pebax + 20 wt.% of Mo-ZeY	3.29±0.21	6.188
